# Optimal strategies to screen health care workers for COVID-19 in the US: a cost-effectiveness analysis

**DOI:** 10.1186/s12962-021-00336-x

**Published:** 2022-01-15

**Authors:** Sigal Maya, Guntas Padda, Victoria Close, Trevor Wilson, Fareeda Ahmed, Elliot Marseille, James G. Kahn

**Affiliations:** 1 University of California San Francisco, San Francisco, CA USA; 2 University of Vermont College of Medicine, Burlington, VT USA; 3 San Joaquin General Hospital, French Camp, CA USA; 4 Stanford University Graduate School of Business, Stanford, CA USA; 5 Health Strategies International, Oakland, CA USA

**Keywords:** Covid-19, Sars-cov-2, Health care workers, Cost-effectiveness, Screening

## Abstract

**Background:**

Transmission of SARS-CoV-2 in health care facilities poses a challenge against pandemic control. Health care workers (HCWs) have frequent and high-risk interactions with COVID-19 patients. We undertook a cost-effectiveness analysis to determine optimal testing strategies for screening HCWs to inform strategic decision-making in health care settings.

**Methods:**

We modeled the number of new infections, quality-adjusted life years lost, and net costs related to six testing strategies including no test. We applied our model to four strata of HCWs, defined by the presence and timing of symptoms. We conducted sensitivity analyses to account for uncertainty in inputs.

**Results:**

When screening recently symptomatic HCWs, conducting only a PCR test is preferable; it saves costs and improves health outcomes in the first week post-symptom onset, and costs $83,000 per quality-adjusted life year gained in the second week post-symptom onset. When screening HCWs in the late clinical disease stage, none of the testing approaches is cost-effective and thus no testing is preferable, yielding $11 and 0.003 new infections per 10 HCWs. For screening asymptomatic HCWs, antigen testing is preferable to PCR testing due to its lower cost.

**Conclusions:**

Both PCR and antigen testing are beneficial strategies to identify infected HCWs and reduce transmission of SARS-CoV-2 in health care settings. IgG tests’ value depends on test timing and immunity characteristics, however it is not cost-effective in a low prevalence setting. As the context of the pandemic evolves, our study provides insight to health-care decision makers to keep the health care workforce safe and transmissions low.

**Supplementary Information:**

The online version contains supplementary material available at 10.1186/s12962-021-00336-x.

## Introduction

In December 2019, a novel zoonotic coronavirus, SARS-CoV-2, emerged in Wuhan, China, and became a global pandemic [1, 2]. With over 82 million confirmed cases and nearly two million fatalities worldwide as of December 30, 2020, it has surpassed the impact of the severe acute respiratory syndrome epidemic of 2002 [3, 4]. In March 2020, outbreaks of COVID-19, the disease caused by SARS-CoV-2, started occurring in the United States. The country soon had the highest number of COVID-19 cases and fatalities worldwide [3, 5].

COVID-19 usually initiates as a lower respiratory infection causing mild to severe pneumonia in most cases and inducing multi-organ systemic effects in some. Asymptomatic infection is common, although uncertainty exists on its prevalence with estimates between 20 and 80% [6, 7]. When undetected, asymptomatic infections increase the likelihood of further transmission, emphasizing the need for widespread screening [7]. Health care workers (HCWs) represent a vulnerable population in the context of COVID-19. Due to the nature of their work, they are at heightened risk of SARS-CoV-2 exposure [8, 9]. Screening HCWs for SARS-CoV-2 infection is important in preventing new infections and deaths both in health care facilities and among community members of HCWs.

One approach to screen for SARS-CoV-2 is to assess the presence of viral RNA using polymerase chain reaction (PCR) tests, which can provide important information on infection status and allow infected HCWs to self-isolate [10]. However, as the pandemic progressed, health care facilities across the US faced shortages of PCR testing supplies [11]. Furthermore, a positive PCR test does not necessarily indicate transmissible virus: individuals infected with SARS-CoV-2 may remain PCR-positive for over a month, long after they have cleared active infection [6, 12].

An alternative to PCR tests for screening purposes is rapid antigen (Ag) tests. While these tests have lower sensitivity than PCR, they can identify individuals who have high enough viral loads to transmit infection [13, 14]. Ag tests are relatively inexpensive ($5) and provide results in 15 min [15].

Finally, while not useful for screening for active infection, antibody tests (specifically tests that detect SARS-CoV-2-specific immunoglobulin G [IgG]) can be administered to identify immune HCWs and inform strategic workforce management decisions. Knowledge of antibody status could allow selective assignment of HCWs who have immunity against SARS-CoV-2 to care for COVID-19 patients, thereby lowering the risk of transmission. Yet, there is limited information on how long immunity lasts or if antibodies reliably assess immunity [16–18].

Variable performance of available tests and complex patterns of biological markers also pose significant challenges to health care officials as they attempt to identify optimal screening strategies [19]. The risks associated with false test results, such as providing false reassurances of immunity (false-positive IgG tests) or unnecessary isolation of HCWs (false-positive PCR or Ag tests), should be considered when developing optimal screening strategies for this population. In this study, we used cost-effectiveness analysis to determine the most effective use of these tests.

While the development of SARS-CoV-2 vaccines have rapidly and significantly changed the context of this pandemic, the subsequent emergence of new SARS-CoV-2 strains highlights the possibility that other SARS-CoV-2 variants may emerge in the future that may not be vaccine-susceptible [20]. In the meantime, HCWs continue to be at risk of contracting the virus as the vaccines are being rolled out [21]. As such, it remains crucial to have measures in place to keep the health care workforce safe and reduce transmissions in the workplace and the community.

## Methods

### Overview

**Fig. 1:**
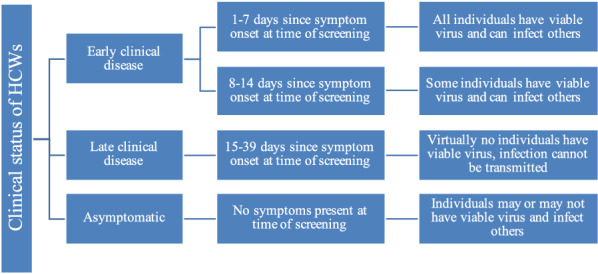
Clinical status of HCWs based on existence of respiratory symptoms at time of screening [22–24]

We conducted a decision model-based cost-effectiveness analysis to identify optimal COVID-19 screening strategies for HCWs. We divided the population into susceptible, infected, and recovered (including vaccinated). To portray the evolution of viral detectability and infectivity through the course of infection, we stratified our population into four groups based on symptom status and duration at time of screening (Fig. 1). Asymptomatic individuals were those without COVID-19 symptoms (i.e., including pre-symptomatic), regardless of history of known or suspected COVID-19 exposure. We designed a decision tree incorporating test performance as well as a set of specified actions based on test results. We estimated and compared the number of new infections acquired, quality-adjusted life years (QALYs) lost, and net costs associated with one-time testing using six testing options: (1) no tests, (2) only PCR test, (3) only Ag test (4) only IgG test, (5) conditional PCR test if IgG test is positive, and (6) concurrent IgG and PCR tests. We calculated incremental cost-effectiveness ratios (ICERs) where appropriate and conducted extensive sensitivity analyses. The model was implemented in Excel® (Office 365, Microsoft Corporation) and used @RISK® (Palisade Corporation, version 7.6.1) software for sensitivity analyses.

### Decision tree design

The decision model incorporates three possible COVID-19 infection states and associated serological profiles, representing the true biological profile of a HCW which is unknown at time of screening (see Additional file 1). Six different screening strategies (including no screening) were considered. We model the probability of receiving positive or negative test results as dictated by test sensitivity and specificity. According to test results, HCWs take different specified actions, yielding varying rates of transmission. We calculate number of new infections acquired, QALYs lost, and net costs incurred for each screening strategy. A simplified version of the decision tree is presented in Additional file 1: Appendix G.

### Key assumptions

We made several assumptions in constructing the model: (1) recovered (including vaccinated) individuals have immunity for COVID-19 of 85% [25] (varied in sensitivity analyses). (2) Individuals who have been infected shed viable SARS-CoV-2 for approximately 8 days post-symptom onset, after which the probability of shedding viable virus decreases significantly. Positive PCR (but nog Ag test) past the duration of infectiousness is due to viral RNA fragments [6, 12, 22, 23]. We explore different durations of shedding viable virus. (3) HCWs who know their viral and antibody status behave differently from those who do not. Those who believe they have recovered take more risks than those with no information. (4) Individuals with SARS-CoV-2 infection cannot acquire a second infection while carrying infectious virus.

### Model inputs

The parameter values used in the model are based on extensive literature reviews and personal communications with experts. To account for uncertainty, inputs were varied widely (Table 1).

**Table 1:** Base-case values and ranges for model inputs

Input	Base-case value	Range	Source
**Test performance**			
IgG test sensitivity	98.1%	89.9–99.7%	US FDA [26]
IgG test specificity	99.6%	99.2–99.8%	US FDA [26]
Ag test sensitivity in early clinical disease, days 1–7	90%	70–95%	Pollock [27]
Ag test sensitivity in early clinical disease (days 8–14), late clinical disease (days 15–39), asymptomatic	70%	50–90%	Pollock [27], Pilarowski [28]
Ag test specificity	99.6%	99.6–100%	Pollock [27], Pilarowski [28]
PCR test sensitivity, symptomatic	95%	67–100%	Shen [29], Tahamtan [30]
PCR test sensitivity, asymptomatic	70%	53–95%	Reddy [31], Yang [32], Wang [33]
PCR test specificity in early clinical disease, days 1–7	99%	60–100%	See Additional file 1: Appendix E for calculation
PCR test specificity in early clinical disease, days 8–14	11%	9–12%	
PCR test specificity in late clinical disease	66%	50–100%	
PCR test specificity when asymptomatic	62%	44–82%	
**Cost inputs**			
Cost of COVID-19 treatment	$3312	$1000–$12,000	Rae [34]
Cost of PCR testing	$51	$20–$120	CMS [35]
Cost of IgG testing	$42	$20–$120	CMS [35], Satyanarayana [36], Cairns [37]
**Viral profiles**			
Likelihood of infectiousness in early clinical disease, days 1–7	89.3%	43.6–97.1%	Wölfel [22]
Likelihood of infectiousness in early clinical disease, days 8–14	7.9%	0.7–36.4%	
Likelihood of infectiousness in late clinical disease	0.0%	0.0–0.8%	
**Antibody profiles**			
Likelihood of having no antibodies in early clinical disease, days 1–7	67.6%	61.7–69.9%	Zhao [38]
Likelihood of having no antibodies in early clinical disease, days 8–14	19.3%	10.4–22.3%	
Likelihood of having no antibodies in late clinical disease	0.1%	0.0–1%	
**Epidemiologic inputs**			
Point prevalence of COVID-19 infection in the community	0.002	0.0005–0.008	California COVID-19 Dashboard [39]
Proportion of population recovered (or vaccinated)	0.47	0.43–0.70	California DPH [40]
Probability of asymptomatic infection	0.4	0.2–0.8	Nishiura [7]
Effective reproduction number with precautions	0.85	0.50–1.5	CMMID [41]
Immunity conferred	85%	50–100%	Hall [25]
QALYs lost due to one COVID-19 infection	0.078	0.05–0.21	Avalon Health Economics [42], Ioannidis [43], Mallapaty [44]

#### Health

Three possible states were defined for true disease status. *Susceptible* individuals were defined as those who had never been infected with SARS-CoV-2, those who had recovered but never developed antibodies, or those who had recovered but whose antibodies have waned. These HCWs could acquire but could not transmit virus. *Infected* individuals were defined as individuals who were actively infected with SARS-CoV-2 and could transmit. They may or may not have produced IgG. *Recovered* individuals were defined as those who have SARS-CoV-2 antibodies, either due to vaccination or a previous infection. This does not necessarily indicate clinical recovery. These HCWs could not transmit SARS-CoV-2 and had a lower probability of acquiring virus than susceptible HCWs. Further details provided in Additional file 1: Appendix A.

Inferred disease status of a HCW was determined by test results and did not always match true disease status due to false positive and false negative results (see Additional file 1: Appendix B). Mismatches between true and inferred status have the potential to increase transmission or incur unnecessary precautions.

Inferences of disease status led to different actions. Infected HCWs were isolated. In health care settings, susceptible HCWs were more likely to be assigned to COVID-19 “cold zones” (no COVID-19 patients) and recovered HCWs were more likely to be assigned to COVID-19 “hot zones” (only COVID-19 patients). In community settings, susceptible HCWs were more likely to take rigorous COVID-19 precautions while recovered or vaccinated HCWs were likely less strict. More can be found in Additional file 1: Appendices C and D).

While we did not explicitly model different infection mitigation measures (e.g., social distancing, mask wearing, or frequent hand washing), the effect of these measures on SARS-CoV-2 transmission was implicitly taken into account through the use of the current effective reproduction number to calculate the number of secondary infections.

#### Efficacy

While COVID-19 PCR tests are reported to have a sensitivity of only about 70% [31–33], this is attributable to the problematic timing of test administration [45]. Indeed, PCR assays are often described as having high sensitivity, usually above 90% [29, 30, 46]. However, if an individual is tested shortly after exposure, a negative result is likely as viral load may be too low to detect [30, 45]. A positive PCR test result is also not necessarily indicative of viable SARS-CoV-2 [12, 22, 23]. Herein, sensitivity was estimated as 95% and did not vary by time since symptom onset [29, 30]. PCR sensitivity for asymptomatic HCWs was 70% to account for false negative results early in the infection when the individual does not have sufficient viral load [31–33]. For specificity, we used data on the ability to grow viral cultures to estimate the likelihood of having viable virus at different timepoints post-symptom onset [24]. Specificity of PCR testing was varied between 11 and 99% based on the probability of detecting viable SARS-CoV-2 as opposed to viral RNA fragments. Specificity was calculated as 62% on average for asymptomatic individuals (see Additional file 1: Appendix E).

Specifications of the Ag and IgG tests were determined via literature review and are shown in Table 1.

#### Costs

Testing costs for both IgG and PCR tests include cost of testing supplies (swabs, chemical reagents) and human resource costs. The PCR test cost $51 per test and the IgG test was $42, while the Ag test cost $5 [35]. Our base-case assumed that tests would be conducted in-house where equipment and trained personnel is readily available. Costs could differ if testing is outsourced; this was assessed in sensitivity analyses.

COVID-19 treatment costs differ greatly depending on disease severity. Some infected individuals remain asymptomatic and generate no costs, others may require ambulatory or critical care. We used a weighted mean of costs incurred with varying levels of care [34] (see Additional file 1: Appendix F).

### Model outputs

The number of new infections over two weeks was calculated for each probability path using the likelihood of transmission in health care and community settings given different actions taken by HCWs. An indirect transmission multiplier was set as two to account for the remaining infections in the chain.

The number of QALYs lost are calculated for each strategy. QALYs are a standardized measure incorporating morbidity and mortality due to a disease or condition. We used an estimate of 5.52 discounted QALYs lost due to one COVID-19 death (based on US life tables and age distribution of COVID-19 mortality, discount rate 3%) [42, 47], multiplied by an infection fatality rate estimate of 0.5% [43, 44]. To account for the growing evidence of long-term morbidity, we added an estimated 0.05 additional QALYs per infection [48–51]. We investigated the effect of differing these values in sensitivity analyses.

Net costs included screening and medical costs and were not discounted since all costs were incurred in year 1. Medical costs associated with long-term complications were not included due to a paucity of data; this was investigated in sensitivity analyses.

ICERs, expressed as net costs per QALY saved, were calculated when a strategy had higher costs and better outcomes than another. Strategies associated with lower costs and more QALYs saved were dominant; no ICERs were calculated.

### Sensitivity analyses

Extensive one-way and multi-way sensitivity analyses were conducted. Sensitivity ranges for deterministic analyses were informed by the low and high estimates reported in relevant literature. Monte Carlo simulations with 1000 iterations were run for probabilistic sensitivity analyses, with beta distributions for probabilities. Namely, we addressed the uncertainty around treatment costs, test performance, and prevalence of COVID-19 among HCWs.

## Results

### Base case

We present results separately for asymptomatic HCWs and each of the three clinical periods in which a test may be administered as described in Fig. 1. Major findings are presented in Tables 2, 3, 4, 5, and summarized in Table 6. Further details may be found in Additional file 2.

**Table 2:** Results for screening ten HCWs on day 1–7 of having symptoms

Option	Net cost	∆Costs	QALYs lost	∆QALYs Lost	ICER ($/QALYs)
Only PCR	$4633	n/a	0.09661	n/a	n/a
IgG + PCR	$5037	$404	0.09624	0.00037	$1,081,393
Only Ag	$8293	$3660	0.19313	− 0.09652	Dominated
IgG, if positive PCR	$64,297	$59,664	1.49459	− 1.39798	Dominated
No test	$82,172	$77,539	1.92529	− 1.82868	Dominated
Only IgG	$82,497	$77,863	1.92305	− 1.82644	Dominated

Almost all infected individuals have viable virus at this time and positive PCR test results are treated as true positives, indicating isolation

Ag: antigen; ICER: incremental cost-effectiveness ratio; IgG: immunoglobulin G; PCR: polymerase chain reaction; QALY: quality-adjusted life year; ∆: difference

**Table 3:** Results for screening ten HCW on day 8–14 of having symptoms

Option	Net cost	∆Cost	QALYs lost	∆QALYs lost	ICER ($/QALYs)
Only Ag	$353	n/a	0.00709	n/a	n/a
Only PCR	$560	$207	0.00117	0.00592	$34,980
IgG, if positive PCR	$836	$277	0.00860	− 0.00743	Dominated
IgG + PCR	$979	$419	0.00116	0.00001	$34,048,150
No test	$981	$421	0.02299	− 0.02182	Dominated
Only IgG	$1393	$833	0.02279	− 0.02163	Dominated

Some infected individuals have viable virus at this time and positive PCR test results are treated as true positives, indicating isolation

Ag: antigen; ICER: incremental cost-effectiveness ratio; IgG: immunoglobulin G; PCR: polymerase chain reaction; QALY: quality-adjusted life year; ∆: difference

**Table 4:** Results for screening ten HCW on day 15–39 of having symptoms

Option	Net cost	∆Cost	QALYs lost	∆QALYs lost	ICER ($/QALYs)
No Test	$11	n/a	0.00027	n/a	n/a
Only Ag	$61	$49	0.00025	0.00001	$3,909,046
Only IgG	$431	$420	0.00026	0.00001	$37,917,445
IgG, if positive PCR	$483	$472	0.00026	0.00001	$42,650,284
Only PCR	$522	$511	0.00028	− 0.00001	Dominated
IgG + PCR	$941	$930	0.00026	0.00001	$84,011,883

No infected individuals have viable virus at this time and positive PCR test results are treated as false positives; does not indicate isolation

Ag: antigen; ICER: incremental cost-effectiveness ratio; IgG: immunoglobulin G; PCR: polymerase chain reaction; QALY: quality-adjusted life year; ∆: difference

**Table 5:** Results for screening ten asymptomatic HCWs

Option	Net cost	∆Cost	QALYs lost	∆QALYs lost	ICER ($/QALYs)
Only Ag	$86	n/a	0.00084	n/a	Dominant
No Test	$104	$18	0.00244	− 0.00160	Dominated
Only IgG	$523	$437	0.00242	− 0.00158	Dominated
Only PCR	$543	$457	0.00078	0.00006	$7,746,741
IgG, if positive PCR	$704	$618	0.00137	− 0.00053	Dominated
IgG + PCR	$963	$877	0.00078	− 0.00007	$13,370,356

Infected individuals may or may not have viable virus and positive PCR test results are treated as true positives, indicating isolation

Ag: antigen; ICER: incremental cost-effectiveness ratio; IgG: immunoglobulin G; PCR: polymerase chain reaction; QALY: quality-adjusted life year; ∆: difference

**Table 6:** Summary of results

Clinical status	Base case result	Uncertainty
Early clinical period, days 1–7	Only PCR, dominant	PCR testing is 74% likely to save QALYs but only 26% likely to save costs due to variations in test sensitivities
Early clinical period, days 8–14	Only PCR, $34,000/QALY gained	PCR-only is 34% likely to be dominant over Ag testing, if transmissible infection persists into second week post-symptom onset. As this duration decreases, cost-effectiveness of PCR testing also decreases, but remains below $180,000/QALY gained with 50% likelihood
Late clinical period	No test, dominant	No other testing strategy is cost-effective. The magnitude of ICERs depend on QALYs lost per infection and transmission rate of SARS-CoV-2
Asymptomatic	Only Ag, dominant	25% likelihood of being cost-effective (rather than dominant), depending on prevalence of transmissible infection among asymptomatic HCWs and medical costs

*Ag* antigen, *ICER* incremental cost-effectiveness ratio, *PCR* polymerase chain reaction, *QALY* quality-adjusted life year

#### Early clinical period, days 1–7

For ten HCWs who have started experiencing symptoms in the past 7 days, conducting no screening results in 24.8 new infections and 1.925 QALYs lost, generating a net cost of $82,000. This is the least effective option, and it is dominated by PCR-only testing which identifies and mandates isolation for infectious HCWs (Table 2).

Administering only a PCR test is cheaper and more effective than all other strategies except for IgG + PCR testing. While IgG + PCR testing has a small health benefit over only PCR testing, this benefit is so small that the ICER is over $1 million per QALY gained. As such, PCR-only testing is preferred to IgG + PCR, and strictly dominant over the remaining options. For every ten HCWs that are tested with PCR only, the net cost is $4600 with estimated 1.24 new infections, or 0.097 QALYs lost (Table 2). PCR testing only also results in 0.032 HCWs unnecessarily being taken off the health care workforce for 2 weeks for every ten HCWs that are tested during their first week of symptoms.

#### Early clinical period, days 8–14

Results for screening HCWs in their second week post-symptom onset resemble those of HCWs who have been symptomatic more recently, as explained above. Since individuals are more likely to clear infectious virus by this time, the number of new infections and associated costs are significantly lower across all screening approaches compared to HCWs being screened earlier in the course of infection (Table 3). At this stage PCR testing is no longer dominant, but it is the most cost-effective strategy with an ICER of $35 thousand per QALY gained.

#### Late clinical period

Not screening ten HCWs in later stages of clinical disease leads to 0.003 additional infections and 0.0003 lost QALYs, and generates a net cost of $11 due to susceptible HCWs acquiring SARS-CoV-2 (Table 4). At this stage, no test is the dominant strategy. While testing strategies that include Ag or IgG tests provide some minor health benefits compared to no test, these benefits are so small that the cost per QALY gained for all these options are in the order of millions, thus they are not cost-effective. PCR-only testing slightly increases the number of QALYs lost compared to no test due to susceptible HCWs receiving false positive test results and assuming they are protected from infection.

#### Asymptomatic

Not screening asymptomatic HCWs leads to 0.03 new infections, 0.002 QALYs lost and $104 in net costs per ten HCWs (Table 5). Administering an Ag test is the dominant approach for asymptomatic screening costing $86 per ten HCW tested. Both PCR-only and IgG + PCR screening are slightly more effective than Ag-only, however they have significantly higher net costs leading to large ICERs of $8–13 million per QALY gained.

### Sensitivity analyses

**Fig. 2:**
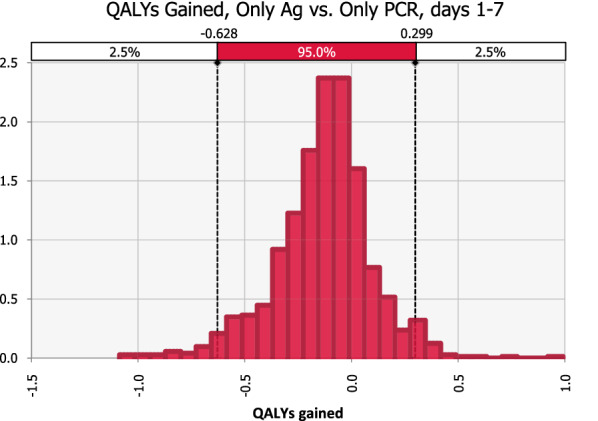
Probability distribution of QALYs saved with Ag vs. PCR-only testing in early clinical disease, days 1–7. PCR screening saves more QALYs than Ag testing in 74% of simulations

**Fig. 3:**
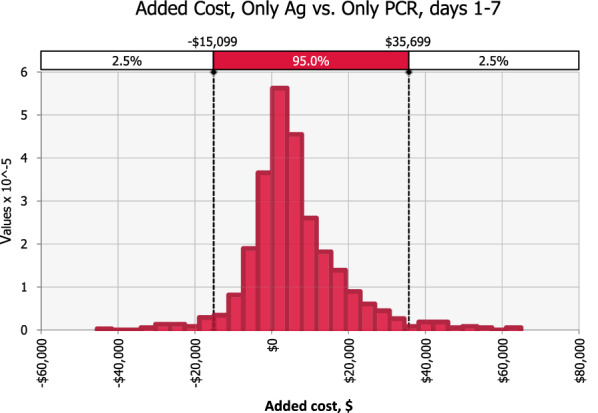
Probability distribution of difference in net costs with Ag vs. PCR-only testing in early clinical disease, days 1–7. PCR screening has fewer net costs than Ag testing in 26% of simulations

Sensitivity analyses reveal little variation in outcomes with changing inputs. For HCWs in the first week of having symptoms, PCR testing was 74% likely to save more QALYs than Ag testing, but only 26% likely to be cheaper (Figs. 2 and 3). Both of these outcomes were most dependent on PCR and Ag test sensitivity which accounted for 45% to 29% of variance.

When testing in the second week, PCR testing remained the preferable strategy but with decreasing cost-effectiveness (ICERs up to $750 thousand per QALY saved), primarily due to the prevalence of transmissible infection in this group which accounted for 23–32% of variance.

In the late clinical disease stage, Ag testing always saved QALYs compared to no test but was not cost-effective due to very small health gains. PCR-only testing in this group had a 12% probability of saving QALYs compared to no testing (as opposed to increasing health losses in base-case), however these gains also were not large enough to render PCR cost-effective over no testing. Thus, no testing remained the optimal strategy. In all three groups of symptomatic HCWs, IgG + PCR testing led to small QALY gains that were dominated by others. Remaining strategies that did not include viral tests (either PCR or Ag) were dominated in simulations.

**Fig. 4:**
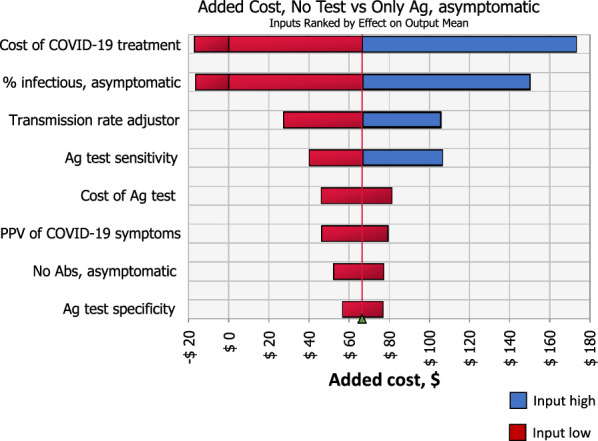
One-way sensitivity analyses on net costs of no test vs. Ag testing among asymptomatic HCWs

For asymptomatic screening, Ag tests always saved QALYs compared to no testing but was more expensive in 25% of simulations when COVID-19 treatment costs or prevalence was low (Fig. 4). In these cases, Ag testing was cost-effective with ICERs up to $80 thousand per QALY gained (as opposed to dominant in base-case). Inputs that most affected this ICER were cost of COVID-19 treatment and prevalence of transmissible infection among asymptomatic individuals, which accounted for 41% and 19% of variance, respectively. Comparing PCR-only to Ag testing, the former had a 65% probability of saving QALYs over Ag testing, however was not cost-effective due to small health gains and large increases in net costs.

## Discussion

This study assessed the cost-effectiveness of six screening approaches for HCWs in the US using one-time PCR, Ag, and/or IgG assays under varying conditions based on clinical presentation. When the prevalence of COVID-19 is low, findings suggest that the best screening approach for recently symptomatic HCWs is PCR testing. Symptomatic HCWs in the first or second week, if truly infected with SARS-CoV-2, are more likely to be infectious than those in the later stages of clinical disease, and isolating these individuals is more important in reducing transmission than knowing their antibody status. Notably, studies have shown Ag test sensitivity approaches that of PCR when testing those within first week of symptoms [27, 28]. In this case, Ag testing might be a promising alternative for screening for its lower cost and faster turnaround time. Indeed, our sensitivity analyses show variable health gains and cost savings with these two tests, suggesting the possibility of Ag testing being dominant (cheaper and more effective) over PCR-only.

Coupling PCR testing with an IgG test provides a minute health gain due to fewer susceptible HCWs being assigned to COVID-19 “hot zones” where risk of acquiring infection is higher. Yet, IgG + PCR testing is not a cost-effective option, as the additional net cost of this strategy is prohibitive.

Conversely, for HCWs in late clinical disease, the risk of still being infectious is low, and knowledge of antibody status is more helpful to reduce transmission by strategic workforce management if long-lasting immunity is conferred. As the course of the infection progresses, IgG titer levels increase. As such, its utility increases over time. Previous studies on antibody responses to SARS-CoV-2 also recommend conducting IgG tests around the third week of infection due to higher detection rates at this time [38]. Yet, with ICER’s over $30 million per QALY gained, strategies incorporating IgG tests are still not cost-effective in late clinical disease because of the low prevalence of COVID-19. Notably, PCR testing in late clinical disease leads to poorer health outcomes than no screening at all. A positive PCR result for HCWs who are three or more weeks post-symptom onset is interpreted as a false positive (i.e., due to viral fragments, not transmissible). These individuals are falsely reassured that they have recovered from COVID-19 and therefore are protected. They may have lower fidelity to personal protective equipment and may take more risks, leading to a slight increase in the number of new infections. A positive Ag test is not interpreted as a false-positive at this stage (even though it may be), because unlike PCR tests, Ag test are not suspect of detecting low and non-transmissible viral loads.

For screening asymptomatic HCWs, Ag testing is preferable over others. It is cheaper and more effective than most other testing strategies. PCR-only testing saves additional QALYs, however the high cost of PCR compared to Ag tests leads to a high ICER that is not cost-effective. Testing strategies that include antibody testing are all dominated by PCR-only testing, because false positive antigen tests of asymptomatic HCWs lead to false reassurances of protection and more risk-taking.

Findings of robust memory T cell responses to SARS-CoV-2 in the absence of antibodies suggest that seronegative individuals may also be protected from reinfection [52]. This emphasizes the possibility of controlling transmission in health care settings by strategic decision-making. However, it also indicates that the proportion of HCWs who are immune might have been underestimated in this study, leading to underestimated cost-effectiveness of all screening strategies. Different ways of assessing immunity may increase effectiveness of strategic workforce management in lowering transmissions of SARS-CoV-2 in health care settings.

Our analysis provides insight for health care decision makers. We highlight the importance of scaling up PCR and Ag testing to identify infectious HCWs to reduce spread of infection in health care and community settings. We also demonstrate the potential benefits of strategic scheduling of HCWs while noting that long-term seroprevalence and immunological studies are needed before policy recommendations can be given with confidence. While this analysis does not find antibody testing cost-effective for HCWs, this strategy does allow reductions in transmission to and from HCWs in late clinical disease. This suggests that alternative ways of assessing immunity may provide a cost-effective approach to informing workforce management decisions to reduce spread.

### Limitations

Uncertainty, while always an issue in cost-effectiveness analyses, is particularly a concern here, given the limited epidemiological and clinical information on COVID-19. We therefore made several assumptions based on characteristics of other similar pathogens, such as SARS, and used informed estimates for data inputs. Specifically, the definition we used for PCR specificity based on infectivity rather than being infected introduced some uncertainty. To address this limitation, we consulted with experts between March–July 2020 and conducted sensitivity analyses using wide ranges for these inputs. We found that varying these key inputs did not substantially change findings.

Second, the simulated IgG test provides a quantitative measure of IgG and does not differentiate between neutralizing and non-neutralizing antibodies. The immunologic ramifications of having IgG are also not yet fully understood. Our base-case analysis assumes high protection but does not make any conjectures about duration of immunity. If IgG provides transient protection, then the falsely reassuring effect of IgG testing across all four strata of HCWs would increase over time.

Third, this study does not incorporate the loss of productivity due to unnecessary isolation of HCWs. It is plausible that given this inefficiency, PCR testing may not be a dominant screening approach. Nonetheless, PCR testing would remain the most effective strategy to decrease the number of new infections because it would still identify the most cases. Studies assessing screening strategies for the general workforce and college campuses corroborate that PCR screening is a cost-effective option compared to options such as symptom or fever screening [53, 54].

Fourth, the utility of Ag testing is debated. The test’s analytical sensitivity is poor, while it can more accurately identify higher viral loads which are more likely to be transmissible. This suggests that our model underestimates the cost-effectiveness of Ag testing for asymptomatic HCWs, and that it is an even more preferable strategy for this group than we estimate.

Finally, we recognize that some oversimplifications were made. The decisions we modeled are based solely on test results, whereas in reality these test results would be considered alongside exposure history among other factors that may influence health outcomes.

While these uncertainties are real, we believe that our assumptions and estimates reflect the best available evidence at the time of this writing. As new information becomes available, the interpretation of our results will likely change.

### Future work

There is much to be discovered regarding SARS-CoV-2 and COVID-19, and new findings must be integrated into this analysis to improve the accuracy of inputs and results. If other testing approaches become available, these should considered in the model. This analysis reflects cost-effectiveness of screening strategies in the US where the prevalence of COVID-19 is low. The results and their interpretation will differ in other settings where conditions may be different. As the pandemic moves through more vulnerable regions, it will be critical to apply this analysis to different populations to help ensure that critical resources are allocated optimally.

## Conclusion

As a result of the COVID-19 pandemic, resource shortages occurred throughout the US. We conducted a cost-effectiveness analysis to understand the optimal allocation of testing resources for COVID-19 to screen HCWs and to inform workforce management decisions. Our results suggest that the screening approach should be different depending on the clinical presentation of HCWs being screened. Among the testing strategies analyzed, PCR testing is the dominant approach for HCWs who started seeing COVID-19 symptoms in the past 1–14 days, whereas Ag tests should be preferred for asymptomatic HCWs. For those who are more than 15 days post-symptom onset, PCR testing has low utility and IgG testing is too expensive; no testing is the optimal approach. These findings are based on US-specific inputs and several key assumptions. As the body of evidence grows, these findings should be reviewed and updated.

## Supplementary Information

**Additional file 1.** Additional information on methods.

**Additional file 2.** Additional information on results.

## Data Availability

All data generated or analysed during this study are included in this published article and its Additional files.

## Abbreviations

Ag: Antigen
COVID-19: Coronavirus disease 2019
HCW: Health care worker
ICER: Incremental cost-effectiveness ratio
IgG: Immunoglobulin G
PCR: Polymerase chain reaction
QALY: Quality-adjusted life year
SARS-CoV-2: Severe acute respiratory syndrome coronavirus 2
